# Follow-up after the Ross procedure. How significant it is

**DOI:** 10.1186/1749-8090-10-S1-A274

**Published:** 2015-12-16

**Authors:** Panagiotis Artemiou, Ingrid Schusterova, Frantisek Sabol

**Affiliations:** 1 Dept. of Cardiac Surgery, University of J P Safarik, Eastern Slovakia Institute for Cardiovascular Diseases, Kosice, Slovakia; 2 University of J P Safarik, Children's Faculty Hospital, Kosice, Slovakia

## Background/Introduction

Replacement of the aortic valve or aortic root with a pulmonary autograft (Ross procedure) and replacement of the pulmonary valve with a pulmonary allograft or xenograft and is now a widely used technique for the treatment of aortic valve disease in the children and young adult. Excellent short and midterm results have been demonstrated but there is continuing concern about the risk of long-term autograft dilation and aortic regurgitation.

## Aims/Objectives

Case report 1

A 24 year old female patient in 2002 at the age of 12 underwent a Ross procedure due to severe congenital aortic valve stenosis. During the follow-up, in the last year the aortic root dilated from 30 mm to 34 mm. Other findings were mild aortic regurgitation and mild pulmonary valve stenosis. The patient is still under surveillance and clinically is on NYHA class II.

## Method

Case report 2

A 29 year old female patient in 2002 at the age of 17 underwent a Ross procedure due to a congenital severe aortic valve stenosis combined with regurgitation. During the follow-up the aortic root and the ascending aorta gradually dilated to 52 mm and 50 mm respectively with also mild to moderate aortic regurgitation.

In 2014 she underwent a redo-Bentall operation with the replacement of the dilated aorta and aortic valve with a mechanical conduit ATS 22 mm/26 mm (Medtronic Inc MN USA). After the operation the patient is stable and is doing well.

## Results

Case report 3

A male patient in the year 2000 at the age of 10 underwent a Ross procedure due to severe aortic valve regurgitation combined with stenosis. During the operation also annuloplasty of the aortic ring was done because of the difference between the native aortic ring and the pulmonary autograft diameters. In 2007 he underwent balloon dilation of the neoartic valve due to severe stenosis with a decrease of the peak gradient from 80 mmHg to 30 mmHg. During the follow-up aortic root and ascending aorta gradually dilated to 50 mm and 56 mm respectively. Moreover the aortic stenosis after the balloon dilatation also deteriorated. In 2010 the patient was referred to surgery for replacement of the aortic root and the ascending aorta. Unfortunately, the patient died from aortic rupture before surgery during the preoperative evaluation.

## Discussion/Conclusion

In conclusion, dilatation of the pulmonary autograft is a major drawback of the Ross procedure and it is the leading cause for reoperation. In order to prevent any lethal or non-lethal complications of the pulmonary autograft these patients need a close and life-long systematic follow-up.

## Consent

Written informed consent was obtained from the patient for publication of this abstract and any accompanying images. A copy of the written consent is available for review by the Editor of this journal.

